# PSI/Masc-Dsx Regulatory Network in Silkworm Sex Determination Pathway

**DOI:** 10.3390/insects17070742

**Published:** 2026-07-20

**Authors:** Chengya Tan, Dongbin Chen, Shuai Zhang, Huiling Dai, Yang Ye, Bingpeng Liu, Hui Lu, Jun Xu

**Affiliations:** 1 Jiangsu Key Laboratory of Sericultural and Animal Biotechnology, School of Biotechnology, Jiangsu University of Science and Technology, Zhenjiang 212100, China; 232211202117@stu.just.edu.cn; 2 Key Laboratory of Silkworm and Mulberry Genetic Improvement, Ministry of Agriculture and Rural Affairs, Sericultural Scientific Research Center, Chinese Academy of Agricultural Sciences, Zhenjiang 212100, China; 3 State Key Laboratory of Plant Trait Design, Center for Excellence in Molecular Plant Sciences, Chinese Academy of Sciences, Shanghai 200032, China; dbchen@cemps.ac.cn (D.C.); zhangshuai23@cemps.ac.cn (S.Z.); daihuiling24@cemps.ac.cn (H.D.); yeyang25@cemps.ac.cn (Y.Y.); bpliu@cemps.ac.cn (B.L.); hlu@cemps.ac.cn (H.L.)

**Keywords:** *Bombyx mori*, *PSI*, *Masc*, *dsx*

## Abstract

In the silkworm, *Bombyx mori* (Lepidoptera: Bombycidae), the sex determination cascade is regulated by the cooperative actions of *P-element somatic inhibitor* (*BmPSI*) and *Masculinizer* (*BmMasc*), which control the sex-specific splicing of *doublesex* (*Bmdsx*). However, the functional interplay between these two critical factors remains poorly understood. In this study, we investigated the cooperative roles of *BmPSI* and *BmMasc* through transgenic overexpression and pull-down assays coupled with mass spectrometry. Co-overexpression of *BmPSI* and *BmMasc* resulted in female-specific lethality, whereas overexpression of either gene alone caused no lethality. Pull-down coupled with mass spectrometry identified 52 common interacting proteins of BmPSI and BmMasc, including Lethal(3)neo18, a mitochondrial respiratory chain Complex I core subunit, suggesting a potential link between sex determination and mitochondrial function. Transcriptome analysis of previously generated *Bmdsx* knockout mutants (*Bmdsx^M^*, *Bmdsx^F^*, and *Bmdsx^C^*) revealed 703 commonly dysregulated genes, which were predominantly expressed in gonads and enriched in cytoskeletal and metabolic pathways. Notably, the oxidative phosphorylation pathway was enriched in both the *Bmdsx* mutant transcriptome and the BmPSI/BmMasc common interactome. Collectively, these findings indicate that BmPSI and BmMasc cooperate to regulate sex determination, and raise the possibility that synergistic disruption of mitochondrial function may underlie the female-specific lethality observed upon their co-overexpression. This study provides new insights into the molecular crosstalk between sex determination and mitochondrial metabolism in insects.

## Simple Summary

The silkworm, *Bombyx mori* (Lepidoptera: Bombycidae), is not only an important insect for silk production but also a model organism for studying insect sex determination. Elucidating the genetic mechanisms that govern sexual development in the silkworm can facilitate the improvement of breeding and pest management strategies in related species. In this study, we investigated how two key genes, *PSI* and *Masc*, collaborate to regulate sex determination. We found that simultaneous overexpression of both genes leads to female-specific lethality, while males remain unaffected. Through protein interaction analysis, we discovered that these two gene products interact with a protein involved in mitochondrial energy production, suggesting a functional link between sex determination and cellular energy metabolism. Furthermore, we identified a set of genes regulated by *doublesex*, the terminal master switch in the sex determination pathway, many of which are associated with cell structure and metabolism. Collectively, our findings suggest a potential link between upstream sex determination regulators and mitochondrial function, providing new insights into how sex determination pathways may modulate cellular metabolism to influence sex-specific development in insects.

## 1. Introduction

The mechanisms of insect sex determination vary enormously across species, the primary signals are highly diverse, whereas the downstream regulators remain largely conserved [1,2]. Generally, the insect sex determination cascade consists of three levels. First, a highly variable primary signal determines sexual fate during early embryogenesis. Subsequently, regulatory factors transmit this signal to the conserved terminal effector, *doublesex* (*dsx*), which ultimately controls sexual differentiation through sex-specific alternative splicing [3,4,5]. In the model organism *Drosophila melanogaster*, the X chromosome dosage (X: A ratio) serves as the primary signal and hierarchically regulates sex determination through *Sex-lethal* (*Sxl*), *transformer* (*tra*), *transformer-2* (*tra2*), culminating in the sex-specific splicing of *dsx* [6,7,8]. Female flies carry two X chromosomes, which activate *Sxl* transcription. *Sxl* then directs the female-specific splicing of *tra* mRNA. The resulting Tra protein, together with Tra2, controls the female-specific splicing of *dsx*. In contrast, male flies carry only one X chromosome, which leads to the suppression of *Sxl* expression and ultimately determines male sexual fate [9,10]. However, even within Diptera, the primary signals diverge. *Aedes aegypti* utilizes *Nix*, a distant *tra2* homolog, as its male-determining factor [11]. In the housefly *Musca domestica*, a female-determining allele of the *tra* gene initiates sex determination [12], whereas the medfly *Ceratitis capitata* possesses a Y-linked male-determining factor [13,14]. In other insect orders, *tra* serves as a relatively conserved key regulator. For instance, in honeybees, *Apis mellifera*, the *complementary sex determiner* (*csd*) gene regulates the *tra* ortholog *feminizer* (*fem*), which in turn controls female-specific *dsx* splicing [15,16]. In beetles such as *Tribolium castaneum*, *tra* also plays a similar central role [17,18]. Recent conceptual frameworks have proposed classifying primary sex determination signals into “loop starters” and “loop breakers”, providing a unifying perspective on the evolutionary dynamics of these diverse mechanisms [1].

Most lepidopteran insects possess a ZW/ZZ sex determination system. In the silkworm *Bombyx mori*, for example, the female is the heterogametic sex (ZW), while the male is homogametic (ZZ) [4,19,20]. A PIWI-interacting RNA (piRNA) precursor, *Feminizer* (*Fem*), located on the W chromosome, has been identified as the primary signal for sex determination in *B. mori*. In female silkworms, piRNA derived from *Fem* silences the expression of the Z chromosome-linked *Masculinizer* (*Masc*) gene via the piRNA pathway [21], leading to the female-specific splicing of *dsx* and instructing female development. The endogenous *Masc* transcript contains a cis-regulatory element recognized by the fem piRNA, which mediates this female-specific cleavage [21]. In ZZ individuals lacking *Fem*, Masc protein accumulates, promotes male differentiation, and also regulates dosage compensation [21,22,23]. Similarly, in pyralid moths such as *Ephestia kuehniella* and *Plodia interpunctella*, a W chromosome-derived piRNA named *Mom* (*Moth-overruler-of-masculinization*) performs the same function by targeting *Masc* mRNA [23,24]. Nevertheless, some lepidopterans have evolved mechanisms that do not rely on a W-linked primary trigger. In the butterfly *Bicyclus anynana*, the zygosity of a hypervariable region of the Z-linked *BaMasc* gene determines sex [4,25]. The silkmoth *Samia cynthia ricini* employs a Z-chromosome counting system reminiscent of the *Drosophila* X:A ratio mechanism [4]. The W chromosome in Lepidoptera shows remarkable evolutionary dynamics, with multiple independent origins across different lineages [26]. These findings underscore both the plasticity of primary signals and the central, conserved role of *Masc* at the top of the lepidopteran sex determination cascade.

Departing from the *tra*-centric model observed in many insects, Lepidoptera insects lack a *tra* ortholog and instead employ distinct regulatory factors [4,18,27]. In *B. mori*, two RNA-binding proteins BmPSI (P-element somatic inhibitor) and BmIMP (IGF-II mRNA-binding protein), function downstream of *Masc* as key regulators of male-specific *Bmdsx* splicing. BmPSI binds to the CE1 silencer element within the female-specific exon 4 of *Bmdsx* pre-mRNA, promoting its exclusion in males [10,28,29]. BmIMP enhances the RNA-binding activity of BmPSI, thereby stabilizing the male-specific splicing complex [30]. The functional role of PSI in sex determination appears to be conserved across Lepidoptera. In the diamondback moth *Plutella xylostella*, CRISPR/Cas9-mediated disruption of *PxPSI* caused male-specific defects in genital development and induced male sterility, accompanied by the appearance of female-specific *dsx* transcripts in males [31]. Similarly, the PSI homolog in *Spodoptera litura* was shown to bind the CE1 element of *Sldsx* pre-mRNA in a manner analogous to BmPSI, indicating functional conservation of the PSI/dsx regulatory axis in lepidopteran sex determination [32]. In the hemipteran insect *Bemisia tabaci*, a PSI ortholog has been identified and shown to produce multiple sex-specifically spliced isoforms, suggesting that PSI may play a broader role in insect sex determination beyond Lepidoptera [33]. A recent study demonstrated that BmMasc physically interacts with BmPSI to form a functional protein complex. This complex directly regulates male-specific *Bmdsx* splicing by binding to regions adjacent to the female-specific exons of *Bmdsx* pre-mRNA, thereby coupling the primary sex-determination signal to alternative splicing regulation [34]. In addition to these data collected from the ovarian cell line, further genetic evidence is needed to elucidate how *BmPSI* and *BmMasc* work together in sex determination. As the terminal effector of the sex determination cascade, *dsx* governs the differentiation of sexually dimorphic traits and behaviors [29,35,36,37,38]. Although progress has been made in identifying DSX binding sites and candidate target genes, how *dsx* selectively regulates distinct downstream genes across different tissues and developmental stages remains to be fully clarified [39,40].

Emerging evidence suggests a functional link between mitochondrial function and sex-specific development. Due to the maternal inheritance of mitochondria, natural selection acts predominantly on female mitochondrial function, which is associated with sexually antagonistic effects on fitness traits [41]. In *Drosophila*, mtDNA sequence variation has been shown to exert sex-specific effects on fertility and longevity, with males often more susceptible to mitochondrial dysfunction than females [42]. Furthermore, mitochondrial respiratory chain function is essential for spermatogenesis, and mitochondrial dysfunction disproportionately affects male fertility in several insect species [43]. These observations raise the intriguing possibility that the sex determination machinery may interface with mitochondrial function to modulate sex-specific development and survival, a hypothesis that has not been explored in Lepidoptera.

In the present study, we generated transgenic silkworm lines overexpressing *BmPSI* and *BmMasc*. Overexpression of *BmMasc* alone led to partial female-to-male sex reversal, whereas no obvious phenotype was observed in *BmPSI* overexpression lines. Notably, simultaneous overexpression of both *BmPSI* and *BmMasc* resulted in female-specific lethality. Using pull-down assays coupled with mass spectrometry, we identified 52 common interacting proteins of BmPSI and BmMasc. Among these, the mitochondrial respiratory chain Complex I core subunit Lethal(3)neo18 may be involved in the female-specific lethality phenotype. Additionally, we previously generated *Bmdsx* knockout mutants targeting the female-specific (*Bmdsx^F^*), male-specific (*Bmdsx^M^*), and common (*Bmdsx^C^*) isoforms, and RNA-seq analysis revealed that downstream genes of *Bmdsx* were predominantly associated with cytoskeletal and metabolic pathways. Furthermore, the oxidative phosphorylation pathway was enriched in the *Bmdsx* mutant transcriptome, and the BmPSI/BmMasc common interactome included a core subunit of mitochondrial Complex I (Lethal(3)neo18). This convergence of independent proteomic and transcriptomic evidence suggests a potential link between the sex determination cascade and mitochondrial energy metabolism, a previously unrecognized connection in silkworm biology.

## 2. Materials and Methods

### 2.1. Silkworm Strains and Bmdsx Mutant Alleles

All experiments were conducted using the silkworm strain *B. mori* with a Nistari genetic background (a polyvoltine, non-diapause strain). Larvae were reared on fresh mulberry leaves under standard conditions. The *Bmdsx* mutant alleles (*Bmdsx^F^*, *Bmdsx^M^*, and *Bmdsx^C^*) were used, and their generation and characterization have been described previously [10,44,45]. Briefly, *Bmdsx^F^* mutants exhibit female sterility, *Bmdsx^M^* mutants exhibit male sterility, and *Bmdsx^C^* mutants are completely sterile in both sexes.

### 2.2. Plasmid Construction and Germline Transformation

The *PSI* and *Masc* overexpression plasmids were constructed based on the PXL-*IE1*-DsRed or PXL-*IE1*-GFP-*IE1*-SV40 backbones [10,46]. The fem piRNA-binding sequence of the *Masc* gene was mutated from CAAAAAGAGGTAACAATT (*Masc*-WT) to CAGAAGGAAGTCACGATC (*Masc*-Mu). *PSI* and *Masc* were individually cloned into the PXL-*IE1*-GFP-*IE1*-SV40 overexpression vector to generate PXL-*IE1*-GFP-*IE1*-*PSI* and PXL-*IE1*-GFP-*IE1*-*Masc*-Mu (designated as *PSI-O* and *Masc-O*, respectively). Using the two previously constructed plasmids as templates, the *IE1*-*PSI* and *IE1*-*Masc*-Mu sequences were cloned into the PXL-*IE1*-DsRed vector to generate the PXL-*IE1*-GFP-*IE1*-*PSI*-*IE1*-*Masc*-Mu plasmid (designated as *P/M*). The primers used in this study are listed in Table S1. Each transgenic plasmid was mixed with the piggyBac helper plasmid and microinjected separately into fertilized eggs at the pre-blastoderm stage. G1 adult moths were crossed with wild-type moths, and the presence of the marker gene product was scored in the G1 progeny using a fluorescence microscope (Nikon AZ100, Melville, NY, USA).

### 2.3. Cell Culture

The BmN cell line, derived from *B. mori* ovary tissue [47], was cultured in TC100 insect medium (Gibco, Grand Island, NY, USA) supplemented with 10% fetal bovine serum at 27 °C. Plasmids *PSI-O*, *Masc-O*, and *P/M* were separately transfected into BmN cells using a lipofection reagent (Invitrogen, Carlsbad, CA, USA) according to the manufacturer’s protocol. For transfection, 500 ng of each plasmid was used per well in triplicate in 24-well cell culture plates. Untransfected cells (blank) and empty-vector-transfected cells (control) were included as negative controls. Cells were harvested 96 h post-transfection for gene expression analysis.

### 2.4. Qualitative and Quantitative RT-PCR

Total RNA was extracted from BmN cells or silkworm tissues using TRIzol reagent (Invitrogen, Carlsbad, CA, USA) and treated with RNase-free DNase I (Ambion, Austin, TX, USA). For the lethal *P/M* line, individual larvae were collected at the L1D2 stage before female lethality onset, when all individuals were still alive. At this stage, females and males showed no size dimorphism; therefore, sex was determined by molecular markers. Specifically, DNA was extracted from the same samples used for RNA extraction, and a female-specific gene located on the W chromosome was amplified to identify females (W-positive) and males (W-negative). RNA was extracted separately from females and males, and the sex was further confirmed by the diagnostic *dsx* splicing pattern. First-strand cDNA was synthesized using the Omniscript Reverse Transcription Kit (Qiagen, Hilden, Germany) in a 20 μL reaction mixture containing 1 μg of total RNA. RT-PCR was performed using KOD Plus polymerase (Toyobo, Osaka, Japan) and gene-specific primers. Amplification of the silkworm *ribosomal protein 49* gene (*Bmrp49*) served as a positive control [48]. Quantitative real-time RT-PCR (Q-RT-PCR) was performed using SYBR Green Realtime PCR Master Mix (Thermo Fisher Scientific, Waltham, MA, USA) on an Eppendorf Mastercycler realplex real-time PCR system (Eppendorf, Hamburg, Germany). Gene-specific primers were used for Q-RT-PCR. Standard curves were generated using a 10-fold serial dilution of mixed cDNA as template. The 2^−∆∆Ct^ method [49] of relative quantification was used to examine the Q-RT-PCR results. Quantitative mRNA measurements were performed in three independent biological replicates, and data were normalized to *Bmrp49* mRNA levels. The sequences of all primers utilized are listed in Table S1.

### 2.5. Pull-Down

The *PSI* and *Masc* overexpression silkworm strains contain a FLAG tag. Therefore, a standard pull-down assay was used to identify proteins interacting with PSI and Masc. Experimental material was collected from male silkworms at the pupal stage. Pupae were frozen in liquid nitrogen, ground into powder, and homogenized in pre-chilled lysis buffer (RIPA buffer containing protease inhibitors). FLAG antibody-conjugated resin (Anti-Flag M2 agarose beads, Sigma-Aldrich, Burlington, MA, USA) was used to capture interacting proteins through incubation, washing with PBS, and elution. Successful pull-down was verified by Coomassie Blue staining. For proteomic identification, protein samples were digested with trypsin, and the resulting peptides were analyzed by LC-MS/MS (Q Exactive, Thermo Fisher Scientific). Wild-type silkworm pupal testis tissue (without FLAG tag) was used as a negative control and processed in parallel with the FLAG-tagged samples using the same FLAG antibody-conjugated beads. Proteins detected in the negative control were excluded from the list of candidate interactors. LC-MS/MS raw data were searched against the *B. mori* reference proteome database (uniprot_*Bombyx_mori*_18319_20170727.fasta) using the MASCOT search engine (Matrix Science, London, UK) [50]. Trypsin was selected as the enzyme with a maximum of two missed cleavages allowed. Carbamidomethylation on cysteine was set as a fixed modification, and oxidation on methionine was set as a variable modification. Peptide mass tolerance was set to 20 ppm and fragment mass tolerance to 0.1 Da. Peptide identifications were filtered with a MASCOT ion score ≥ 20, and the false discovery rate (FDR) for both peptide and protein identification was set to ≤1%. Proteins were considered reliably identified if they were supported by at least one unique peptide meeting the above score threshold.

### 2.6. Proteomics Analysis

For each pull-down group, proteins identified from the LC-MS/MS analysis were compiled for subsequent analysis. Proteins identified exclusively in the PSI pull-down samples (and absent in the Masc pull-down and control samples) were classified as PSI-specific; those identified exclusively in the Masc pull-down samples were classified as Masc-specific; and proteins detected in both PSI and Masc pull-down samples (but absent in the control) were classified as common interacting proteins. To visualize the overlap of identified proteins between the PSI and Masc pull-down groups, a Venn diagram was generated. The distribution of identified proteins across different functional categories was illustrated using a pie chart, providing an overview of the proportion of each protein type relative to the total identified proteins. For functional annotation, Gene Ontology (GO) enrichment and Kyoto Encyclopedia of Genes and Genomes (KEGG) pathway enrichment analyses were performed using the OmicShare cloud platform (www.omicshare.com/tools, accessed on 2 December 2025), which utilizes annotation resources including DAVID [51]. A custom background gene set, comprising all annotated genes in the *B. mori* reference genome [52], was used for the analysis. Statistical significance was assessed using a hypergeometric test, and the resulting *p* values were adjusted for multiple testing using the Benjamini–Hochberg method to control the FDR. GO and KEGG terms with a Q value ≤ 0.05 were considered significantly enriched. Visualization of GO and KEGG results was carried out using the OmicShare platform. Protein–protein interaction network analysis of the 52 common interacting proteins was performed using the STRING database (cn.string-db.org, accessed on 2 July 2026) with *B. mori* as the reference organism.

### 2.7. RNA-Seq and Data Analysis

For each genotype (*Bmdsx^M^*, *Bmdsx^F^*, and *Bmdsx^C^*), testis and ovary samples were collected from 30 individuals at day 3 of the fifth instar (six groups in total). Tissues from each sample group were pooled, and total RNA was extracted for library construction and sequencing. Raw sequencing data were processed for quality control, filtering, alignment, and quantification using FastQC, Trimmomatic, Bowtie2, and RSEM, respectively, with reference to the *B. mori* genome database [52,53,54,55]. Gene expression levels were normalized using DESeq2 [56]. Differentially expressed genes (DEGs) were identified with an FDR < 0.05 and an absolute log_2_(fold change) > 1. GO and KEGG enrichment analyses were performed using the OmicShare cloud platform, with the same parameters as described in Section 2.6. The raw RNA-seq data have been deposited in the NCBI Sequence Read Archive (SRA) under BioProject accession number [PRJNA1461226] with SRA run accession numbers [SRR38358540–SRR38358545].

### 2.8. Statistical Analysis

All data are presented as mean ± standard deviation (SD) from at least two independent biological replicates. Statistical comparisons between two groups were performed using unpaired two-tailed Student’s *t*-test. A *p*-value < 0.05 was considered statistically significant. All statistical analyses were performed using GraphPad Prism 10 (GraphPad Software, Boston, MA, USA) for macOS.

## 3. Results

### 3.1. Co-Overexpression of PSI and Masc Leads to Female-Specific Lethality

**Figure 1:**
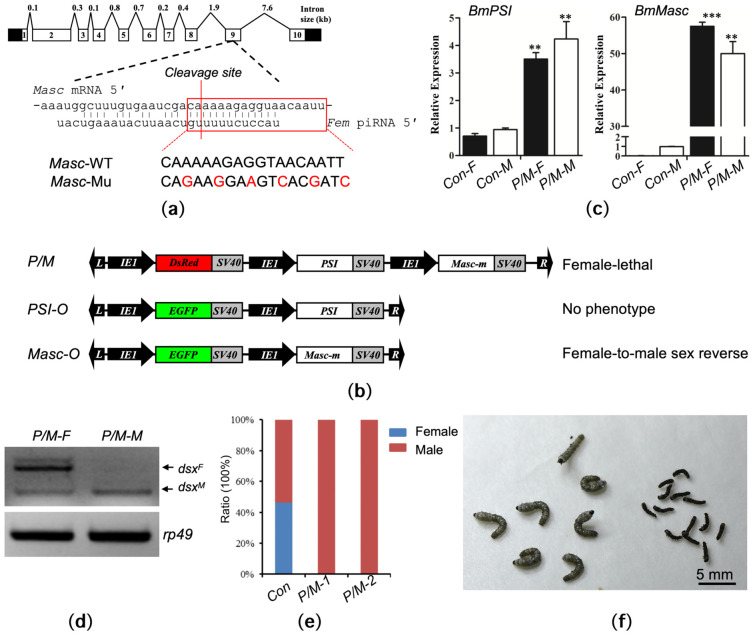
Overexpression of *BmPSI* and *BmMasc* genes induces splicing changes in *Bmdsx* and leads to female lethality. (**a**) Design of mutations in piRNA-binding sites of the *BmMasc* gene. (**b**) Schematic diagrams of the overexpression vectors. (**c**) Relative expression levels of *BmPSI* and *BmMasc* in control and *P/M* lines measured by qRT-PCR. (**d**) RT-PCR analysis of *Bmdsx* splicing patterns. (**e**) Sex ratio statistics of control and *P/M* lines. (**f**) Representative images of *P/M* larvae. Left, normally developed male larvae; right, dead female larvae. Statistical significance: ** *p* < 0.01, *** *p* < 0.001. Abbreviations: Con, control; F, female; M, male; *P/M*, co-overexpression of *BmPSI* and *BmMasc*; *PSI-O*, *BmPSI* overexpression; *Masc-O*, *BmMasc* overexpression; *P/M-1* and *P/M-2*, two independent *P/M* transgenic lines.

To investigate the functions of *BmPSI* and *BmMasc* in the sex determination cascade of *B. mori*, we constructed transgenic lines overexpressing *PSI* (*PSI-O*) and *Masc*-Mu (*Masc-O*), and two independent lines co-overexpressing both genes (*P/M*). To achieve constitutive overexpression of *BmMasc*, we introduced a modified transgene carrying mutations in the cleavage site (Figure 1a). The schematic diagrams of the overexpression vectors are shown in Figure 1b.

To assess the effects on the sex determination pathway, we examined *BmPSI* and *BmMasc* expression levels in two independent *P/M* lines. qRT-PCR analysis showed that both genes were significantly upregulated in both females and males compared with controls (*PSI*: female, *t* = 11.14, df = 2, *p* = 0.0080; male, *t* = 10.64, df = 2, *p* = 0.0087; *Masc*: female, *t* = 52.71, df = 2, *p* = 0.0004; male, *t* = 15.02, df = 2, *p* = 0.0044; unpaired *t*-test) (Figure 1c). We then examined the splicing pattern of the downstream gene *Bmdsx*. Notably, prior to death at the L1D3 stage, *P/M* females exhibited pronounced growth retardation and were smaller than their male siblings. This size dimorphism allowed reliable sex distinction before death. For splicing analysis, we collected *P/M* individuals at an earlier L1D2 stage, before size dimorphism emerged and when all individuals were still alive. Sex was determined by W-chromosome-specific PCR and further confirmed by the diagnostic *Bmdsx* splicing pattern. As shown in Figure 1d, in individuals molecularly identified as females, we detected the female-specific *Bmdsx^F^* isoform (as the predominant band) together with the *Bmdsx^M^* isoform. In contrast, molecularly identified males of the same age exhibited only the *Bmdsx^M^* isoform. These results confirm both the sex of the individuals and that they were sampled while still viable. Analysis of additional cell lines confirmed the specificity of these effects. In control (empty-vector-transfected) and blank (untransfected) cells, only the female-specific *Bmdsx^F^* isoform was detected. Overexpression of *BmMasc* alone (*Masc*-WT or *Masc*-Mu) induced both *Bmdsx^F^* and *Bmdsx^M^* isoforms, while *BmPSI* alone did not alter the splicing pattern and only *Bmdsx^F^* was detected. Co-expression of *PSI* and *Masc*-Mu resulted in both isoforms, with *Bmdsx^F^* slightly more abundant than *Bmdsx^M^* (Figure S1a). qRT-PCR analysis confirmed that *PSI* expression was upregulated in cells transfected with *PSI*-containing plasmids (*PSI-O* and *P/M*), while *BmMasc* expression was increased in cells transfected with *Masc*-containing plasmids (*Masc-O* and *P/M*) (Figure S1b,c). Together, these results demonstrate that co-overexpression of *BmPSI* and *BmMasc* induces ectopic *Bmdsx^M^* activation, an effect dependent on simultaneous upregulation of both genes.

Statistical analysis of the sex ratio revealed that control populations exhibited a balanced ~1:1 sex ratio, whereas two independent *P/M* lines produced 100% male offspring (Figure 1e). Phenotypic observation indicated that the absence of females was attributable to female-specific lethality, as female larvae failed to complete development (Figure 1f).

### 3.2. PSI and Masc Share Common Interacting Proteins Including a Mitochondrial Complex I Subunit

**Figure 2:**
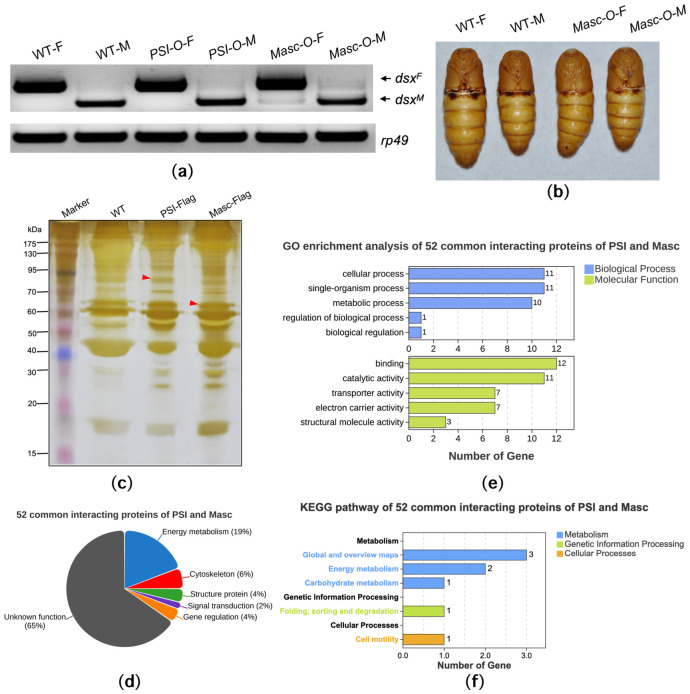
Identification of BmPSI and BmMasc interacting proteins. (**a**) RT-PCR analysis of *Bmdsx* splicing patterns in wild-type, *PSI-O*, and *Masc-O* individuals. (**b**) Representative pupal phenotypes of wild-type and *Masc-O* individuals. (**c**) FLAG pull-down assay. Proteins were separated by SDS-PAGE and visualized by Coomassie Blue staining. Molecular weight markers (kDa) are indicated on the left. Arrowheads indicate the positions of PSI-Flag (~77 kDa) and Masc-Flag (~63 kDa). (**d**) Functional classification of common interacting proteins of PSI and Masc. (**e**) GO enrichment analysis of common interactors. (**f**) KEGG pathway enrichment analysis of common interactors. Abbreviations: WT, wild-type; F, female; M, male.

Given that co-overexpression of *BmPSI* and *BmMasc* resulted in female-specific lethality whereas single overexpression of either gene did not, we hypothesized that a physical interaction between these two proteins may underlie this synergistic effect. We next examined the effects of *BmMasc* overexpression alone. In *Masc-O* females, *Bmdsx* splicing analysis revealed the presence of both *Bmdsx^F^* and *Bmdsx^M^* isoforms, with a faint *Bmdsx^M^* band indicating partial sex reversal (Figure 2a). Consistent with this finding, morphological observation showed that *Masc-O* females exhibited reduced pupal size, resembling males rather than typical females (Figure 2b). This phenotype was consistently observed in all *Masc-O* female pupae examined (*n* > 50 across three independent lines). In contrast, *PSI-O* lines displayed no obvious phenotypic changes.

To explore why co-overexpression of *BmPSI* and *BmMasc* leads to lethality whereas overexpression of either gene alone does not, we performed FLAG pull-down assays using transgenic silkworm strains expressing FLAG-tagged PSI and Masc, followed by mass spectrometry to characterize the captured protein complexes (Figure 2c). Venn diagram analysis identified 261 PSI-specific, 131 Masc-specific, and 52 common interacting proteins (Figure S2a; Table S2). Functional classification showed distinct compositions among these interactor groups. Among the 52 common interactors, 65% were proteins of unknown function, and the remaining 35% were categorized into energy metabolism (19%), cytoskeleton (6%), structure protein (4%), gene regulation (2%), and signal transduction (4%) (Figure 2d). In contrast, PSI-specific interactors were enriched in cytoskeleton (3%), structure protein (2%), translation (8%), energy metabolism (8%), vesicle trafficking (5%), gene regulation (7%), protein folding (6%), and other known functions (8%), with 53% having an unknown function (Figure S2b). Masc-specific interactors showed a distinct profile, with notable proportions in cytoskeleton (7%), structure protein (11%), translation (5%), energy metabolism (3%), signal transduction (6%), gene regulation (4%), protein folding (2%), and other known functions (2%), while 60% were of unknown function (Figure S2c).

GO enrichment analysis of the 52 common interactors indicated that they are primarily involved in biological processes such as cellular processes, single-organism processes, and metabolic processes, with molecular functions enriched in binding and catalytic activity (Figure 2e). KEGG pathway analysis further revealed significant enrichment in metabolic pathways, including global and overview maps, energy metabolism, and carbohydrate metabolism (Q < 0.05, hypergeometric test with Benjamini–Hochberg correction) (Figure 2f). Although 65% of the common interactors are currently annotated as uncharacterized proteins in the silkworm genome database (Table S2), the functional annotation of the remaining 35% consistently pointed to energy metabolism and mitochondrial function, with ‘Energy metabolism’ being the most significantly enriched KEGG category (Q = 0.035). Notably, among the common interactors, we identified a core subunit of the Mitochondrial respiratory chain Complex I-NADH dehydrogenase subunit 5/Lethal(3)neo18, an essential gene whose mutation is known to cause lethal phenotypes (Table S2). To further explore the functional relationships among the 52 common interactors, we performed protein–protein interaction network analysis using the STRING database. The resulting network revealed that approximately one-third of the common interactors are connected by known or predicted interactions, while the remaining proteins appeared as isolated nodes, consistent with the current annotation status of the silkworm proteome (Figure S2d).

### 3.3. Transcriptomic Changes in Gonads of Bmdsx Mutants

**Figure 3:**
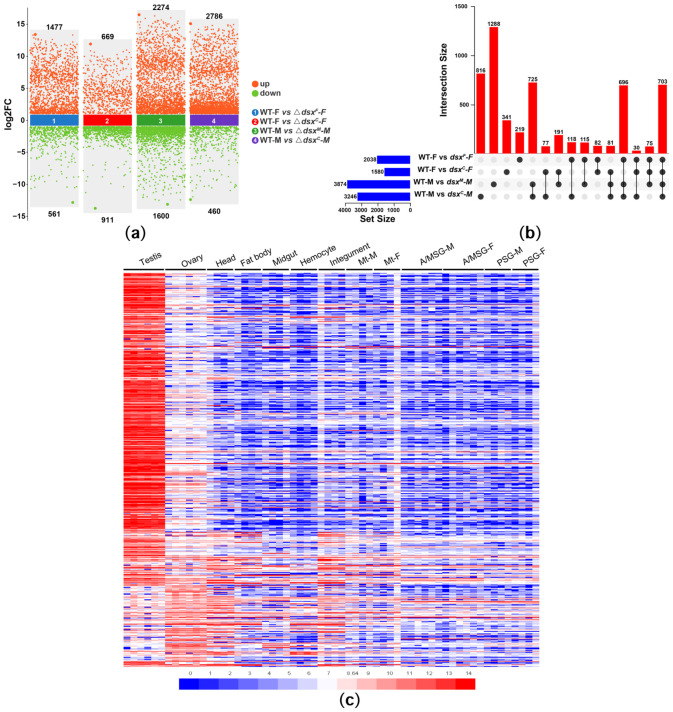
*Bmdsx* knockout causes transcriptomic changes in gonads. (**a**) Numbers of differentially expressed genes in *Bmdsx* mutants. (**b**) UpSet plot of dysregulated genes across four mutant groups. (**c**) Tissue expression profiles of 703 common dysregulated genes across multiple tissues, including testis, ovary, head, fat body, midgut, hemocyte, integument, male Malpighian tubule (Mt-M), female Malpighian tubule (Mt-F), male anterior/middle silk gland (A/MSG-M), female anterior/middle silk gland (A/MSG-F), male posterior silk gland (PSG-M) and female posterior silk gland (PSG-F). Abbreviations: WT, wild-type; F, female; M, male; *Bmdsx^F^*-F, *Bmdsx^F^* mutant (female); *Bmdsx^C^*-F, *Bmdsx^C^* mutant (female); *Bmdsx^M^*-M, *Bmdsx^M^* mutant (male); *Bmdsx^C^*-M, *Bmdsx^C^* mutant (male).

*BmPSI* and *BmMasc* regulate the sex-specific splicing of *Bmdsx*, the terminal effector of the sex determination cascade in *B. mori*. To identify genes downstream of *Bmdsx*, we performed transcriptome sequencing on the *Bmdsx* knockout mutants (*Bmdsx^F^*, *Bmdsx^M^*, and *Bmdsx^C^*). Differential expression analysis revealed widespread transcriptomic perturbations upon *Bmdsx* disruption. In female-sterile mutants, *Bmdsx^F^*-F showed 1477 upregulated and 561 downregulated genes, whereas *Bmdsx^C^*-F exhibited 669 upregulated and 911 downregulated genes. In male-sterile mutants, *Bmdsx^M^*-M displayed 2274 upregulated and 1600 downregulated genes, whereas *Bmdsx^C^*-M showed 2786 upregulated and 460 downregulated genes (Figure 3a). Notably, male-sterile mutants exhibited a substantially higher number of upregulated genes compared with female-sterile mutants. In terms of downregulated genes, *Bmdsx^F^*-F showed 561 and *Bmdsx^C^*-F showed 911, whereas *Bmdsx^M^*-M exhibited 1600 and *Bmdsx^C^*-M exhibited 460, with sex-specific transcriptional responses upon *Bmdsx* loss. An UpSet plot analysis across four comparisons identified 703 genes commonly dysregulated in all four groups (Figure 3b). Tissue expression profiling using the silkworm database revealed that among the 703 commonly dysregulated genes, a substantial portion showed predominant expression in the testis, while a smaller subset exhibited ovary-specific expression, indicating their relevance to reproductive biology (Figure 3c).

We further analyzed sex-specific transcriptional changes. In female-sterile mutants (*Bmdsx^F^* and *Bmdsx^C^*), 890 genes were commonly dysregulated (Figure S3a), with 564 consistently upregulated and 316 consistently downregulated (Figure S3b). GO enrichment analysis of upregulated genes revealed terms associated with extracellular region, cellular component assembly, and microtubule-based processes. KEGG pathway analysis showed enrichment in motor proteins, oxidative phosphorylation, and carbon metabolism. In contrast, downregulated genes were enriched in GO terms related to melanin metabolism and chemosensory behavior, with KEGG analysis revealing downregulation of pathways involved in cell adhesion and signaling, including ECM-receptor interaction and the PI3K-Akt signaling pathway (Figure S3c–f). In male-sterile mutants (*Bmdsx^M^* and *Bmdsx^C^*), 2205 genes were commonly dysregulated (Figure S4a), with 1765 upregulated and 411 downregulated (Figure S4b). GO enrichment analysis of upregulated genes highlighted categories such as catalytic activity, cytoplasm, and microtubule cytoskeleton. KEGG analysis revealed enrichment in motor proteins, oxidative phosphorylation, and carbon metabolism. Downregulated genes were enriched in GO terms related to structural molecule activity and ribonucleoprotein complexes, with KEGG analysis showing downregulation of the ribosome pathway and ECM-receptor interaction (Figure S4c–f).

### 3.4. Commonly Dysregulated Genes Are Enriched in Cytoskeletal and Metabolic Pathways

**Figure 4:**
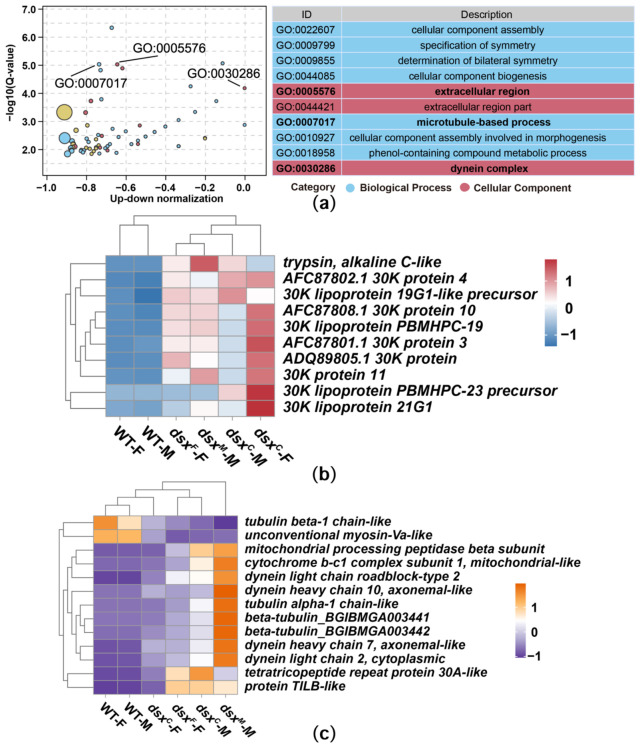
GO enrichment analysis of 703 commonly dysregulated genes in *Bmdsx* mutants. (**a**) Top 10 enriched GO terms (biological processes, cellular components, and molecular functions) ranked by −log10(Q-value). (**b**) Heatmap showing expression patterns of genes enriched in the ‘extracellular region’ pathway. (**c**) Heatmap showing expression patterns of genes enriched in the ‘microtubule-based process’ pathway. Abbreviations are as defined in Figure 3.

To further characterize the functional relevance of the 703 genes commonly dysregulated in all four *Bmdsx* mutants, we performed GO and KEGG enrichment analyses. GO enrichment analysis of these 703 commonly dysregulated genes showed significant enrichment in several biological processes, including cellular component assembly, specification of symmetry, determination of bilateral symmetry, cellular component biogenesis, extracellular region, microtubule-based process, and dynein complex (all Q < 0.05) (Figure 4a). Further examination of the extracellular region pathway showed that most genes in this category were upregulated in all four mutants, with many encoding 30K lipoproteins (Figure 4b). In the microtubule-based process pathway, most genes were also upregulated, except for *tubulin beta-1 chain-like* and *unconventional myosin-Va-like*, which were downregulated in the sterile mutants (Figure 4c). Notably, the dynein complex contained only three genes, which were included in the heatmaps of the other two pathways due to gene overlap.

**Figure 5:**
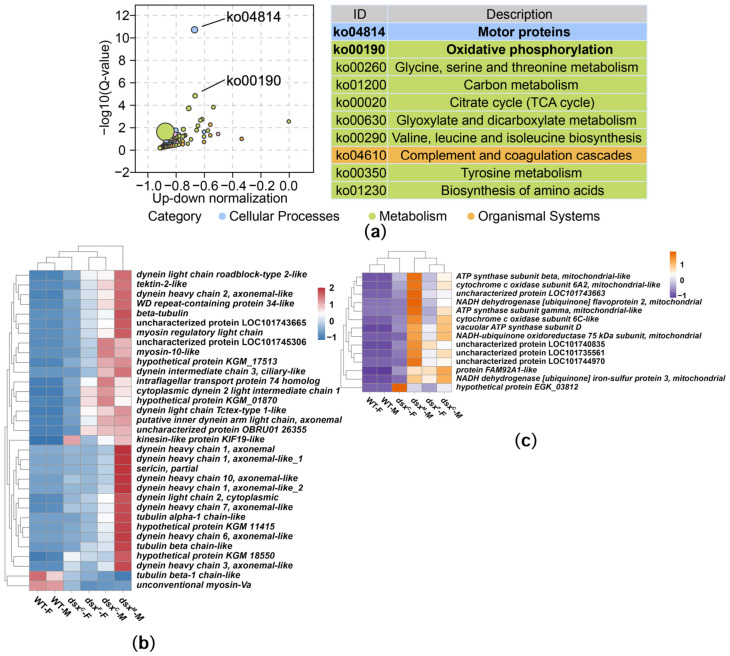
KEGG enrichment analysis of 703 commonly dysregulated genes in *Bmdsx* mutants. (**a**) Top 10 enriched KEGG pathways ranked by −log10(Q-value). (**b**) Heatmap of genes enriched in the ‘motor proteins’ pathway. (**c**) Heatmap of genes enriched in the ‘oxidative phosphorylation’ pathway. Abbreviations are as defined in Figure 3.

KEGG enrichment analysis of the 703 commonly dysregulated genes identified significant enrichment in pathways including Motor proteins, Oxidative phosphorylation, carbon metabolism, the citrate cycle (TCA cycle), and biosynthesis of amino acids (all Q < 0.05) (Figure 5a). Heatmap visualization of genes in the motor proteins and oxidative phosphorylation pathways showed consistent upregulation of most genes across all four mutants (Figure 5b,c). In the motor protein pathway, the dysregulated genes spanned multiple functional categories, including dynein/dynactin complexes, axonemal dyneins, microtubule subunits, kinesins, and myosins, indicating that *Bmdsx* disruption affects diverse cytoskeletal components in the gonads. In the oxidative phosphorylation pathway, the dysregulated genes covered nearly all complexes of the mitochondrial electron transport chain, including Complex I (NADH dehydrogenase), Complex II (succinate dehydrogenase), Complex IV (cytochrome c oxidase), and Complex V (F-type ATPase).

## 4. Discussion

In this study, we found that co-overexpression of *BmPSI* and *BmMasc*-Mu leads to female-specific lethality in *B. mori*, whereas overexpression of either gene alone does not. Through FLAG pull-down combined with mass spectrometry, we identified 52 common interacting proteins of BmPSI and BmMasc, including Lethal(3)neo18, a core subunit of mitochondrial Complex I. Transcriptome analysis of *Bmdsx* knockout mutants further revealed 703 genes commonly dysregulated across sterile mutants, which were enriched in cytoskeletal and metabolic pathways. Notably, the oxidative phosphorylation pathway was enriched in the *Bmdsx* mutant transcriptome and the PSI/Masc common interactome included a core subunit of mitochondrial Complex I (Lethal(3)neo18), suggesting a potential link between sex determination and mitochondrial function.

A recent study demonstrated that BmMasc physically interacts with BmPSI to form a functional protein complex [34]. However, whether additional proteins are involved in the formation of this complex remains unclear. Intriguingly, our study suggested that BmPSI and BmMasc interact with a mitochondrial Complex I subunit. Complex I, the largest complex of the electron transport chain, plays a central role in cellular energy metabolism [57]. In *Drosophila*, disruption of mitochondrial function affects sex-specific development and viability, with mitonuclear interactions exhibiting distinct effects on male and female fitness [58]. These observations are consistent with our FLAG pull-down data showing that BmPSI and BmMasc share multiple common interactors, raising the possibility that they may function together to modulate mitochondrial activity.

In *D. melanogaster*, ectopic expression of *msl2* in females results in assembly of the dosage compensation complex on the female X chromosomes and decreased female viability [59]. Moreover, co-overexpression of both MSL1 and MSL2 in females causes 100% female-specific lethality [60]. In *B. mori*, although the dosage compensation mechanism is less understood, *BmMasc* has been shown to be required for dosage compensation, and its forced expression in females can induce Z chromosome upregulation [21,61]. Therefore, co-overexpression of *BmPSI* and *BmMasc* in females might aberrantly activate the dosage compensation machinery, resulting in female-specific lethality. This hypothesis is consistent with our observation that *P/M* females exhibit develop intellectual disability prior to death. Testing this possibility will require transcriptomic analysis of Z-linked gene expression in pre-lethal *P/M* females, which we plan to undertake in future studies.

We acknowledge that the female-specific lethality observed upon *BmPSI* and *BmMasc* co-overexpression may also be explained by factors other than mitochondrial dysfunction or dosage compensation. For instance, high-level co-expression of two transgenes could induce general cellular stress, which might disproportionately affect females due to sex-specific physiological differences. Alternatively, the observed intellectual disability and lethality could reflect a broader developmental imbalance caused by ectopic activation of male-specific genes (e.g., *Masc*) in a female background, rather than a specific disruption of a single pathway. Furthermore, the FLAG pull-down experiments were performed using overexpressed proteins; therefore, the interaction with the mitochondrial Complex I subunit may be non-physiological or enhanced by supra-physiological protein levels. These alternative interpretations do not diminish the potential interest of the mitochondrial and dosage compensation hypotheses, but they highlight the need for future validation using endogenous co-immunoprecipitation, tissue-specific knockdown, and more direct functional assays (e.g., measurements of mitochondrial respiration) to distinguish among these possibilities.

Although direct evidence linking mitochondrial function to sex determination in the silkworm remains limited, studies in other insects have implicated mitochondria in sex-specific development. In the whitefly *Bemisia tabaci*, mitochondrial function in the ovary influences offspring sex ratios by affecting fertilization [62]. In *Drosophila*, knockdown of the Complex I subunit *ND-42* disrupts mitochondrial derivative maintenance during spermatogenesis, leading to spermatid elongation defects and male sterility [63]. In the silkworm, deletion of *TSSKL* causes severe morphological defects in sperm, accompanied by impaired mitochondrial morphogenesis and male sterility [64]. Together, these findings suggest that mitochondrial function is important for sex-specific reproduction in insects and raise the possibility that *BmPSI* and *BmMasc* may affect mitochondrial function in the silkworm.

Transcriptomic analysis of *Bmdsx* knockout mutants revealed that male-sterile mutants exhibit a substantially higher number of upregulated genes compared with female-sterile mutants. This sex-specific transcriptional response is consistent with previous studies showing that *dsx* regulates distinct target genes in males and females [3,65,66]. In *B. mori*, *Bmdsx* has been shown to control sex-specific splicing of downstream genes involved in reproduction [67,68,69]. Our identification of 703 genes commonly dysregulated across all four mutants, with predominant expression in gonads, further supports the central role of *Bmdsx* in reproductive tissue development. The enrichment of these genes in cytoskeletal and metabolic pathways is consistent with the pleiotropic functions of *Bmdsx* in gonad morphogenesis and germ cell development [68].

The enrichment of cytoskeletal pathways aligns with the critical role of cytoskeletal dynamics in gametogenesis. In the silkworm, disruption of *BmPiezo* causes male sterility accompanied by dysregulation of actin cytoskeleton pathways, underscoring the importance of cytoskeletal integrity for spermatogenesis [70]. The upregulation of oxidative phosphorylation genes across sterile mutants is also noteworthy. While this may represent a compensatory response to metabolic stress, it raises the possibility that *Bmdsx* normally represses these pathways to maintain germ cell development. It is important to note that the RNA-seq data from *Bmdsx* mutants and the protein interaction data from PSI/Masc co-overexpression lines represent two complementary but distinct datasets. The overlap at the level of oxidative phosphorylation pathway enrichment suggests a possible link between these datasets, but it does not establish a direct mechanistic connection between the transcriptomic changes in the *Bmdsx* mutants and the female-specific lethality observed in *P/M* females. Therefore, although the mitochondrial Complex I interaction is an interesting observation, further studies are needed to determine whether and how it contributes to the lethality phenotype.

We acknowledge several limitations in the present study. Functional rescue experiments and direct measurements of mitochondrial function are essential to establish a causal link between mitochondrial dysfunction and the female-specific lethality phenotype, but generating the required transgenic lines and performing the necessary genetic crosses in the silkworm is time-consuming and beyond the current scope. In addition, the enrichment of oxidative phosphorylation in the *Bmdsx* mutant transcriptome and the identification of Lethal(3)neo18 in the PSI/Masc interactome are convergent but correlative, and the upregulation of oxidative phosphorylation-related genes could equally reflect a secondary stress response rather than a primary cause of death. Nevertheless, the convergence of these independent datasets points to a functional link between sex determination and mitochondrial metabolism that warrants further investigation. Future studies directed at assessing mitochondrial functional parameters (ATP, membrane potential, ROS), characterizing the PSI/Masc interaction with Lethal(3)neo18, and performing female-specific knockdown of *Lethal(3)neo18* will be essential to distinguish between these possibilities.

In summary, our findings suggest that BmPSI and BmMasc cooperate to regulate sex determination in *B. mori*. In addition to their known role in controlling *Bmdsx* splicing, they also interact with a mitochondrial Complex I subunit, which may contribute to the female-specific lethality observed upon co-overexpression. Our transcriptomic analysis of *Bmdsx* mutants further showed that *Bmdsx* regulates genes involved in cytoskeletal and metabolic pathways, which are likely important for gonad development and fertility. Together, these results provide new insights into the molecular mechanisms of sex determination and its intersection with mitochondrial function in insects.

## 5. Conclusions

In this study, we demonstrate that *BmPSI* and *BmMasc* cooperate to regulate sex determination in *B. mori*, with co-overexpression causing female-specific lethality. Through proteomic and transcriptomic analyses, we identified mitochondrial Complex I as a common interactor of BmPSI and BmMasc and showed that *Bmdsx* regulates genes involved in oxidative phosphorylation and cytoskeletal pathways. The convergence of independent proteomic and transcriptomic evidence on the oxidative phosphorylation pathway provides cross-omics validation of a previously unrecognized link between the sex determination cascade and mitochondrial energy metabolism in the silkworm, establishing a preliminary foundation for further investigation into how sex determination signals modulate mitochondrial activity to influence sex-specific development and fertility.

## Data Availability

The original contributions presented in this study are included in the article/Supplementary Materials. Further inquiries can be directed to the corresponding author.

## Supplementary Materials

The following supporting information can be downloaded at https://www.mdpi.com/article/10.3390/insects17070742/s1: Figure S1: Validation of overexpression in cultured cells. (a) RT-PCR analysis of *Bmdsx* splicing patterns in the indicated cell samples. (b,c) Relative expression levels of *BmPSI* (b) and *BmMasc* (c) in the indicated cell samples, as determined by qRT-PCR. Statistical significance: * *p* < 0.05, ** *p* < 0.01; ns, not significant. Abbreviations: Control, empty-vector-transfected cells; Blank, untransfected cells; *BmMasc-wt*, wild-type *Masc* overexpression; *BmMasc-m*, mutant *Masc* overexpression; *BmPSI*, *PSI* overexpression; *BmPSI/Masc-m*, co-overexpression of *PSI* and mutant *Masc*; Figure S2: Functional classification of PSI- and Masc-specific interacting proteins. (a) Venn diagram of proteins identified by FLAG pull-down. (b) Functional classification of PSI-specific interacting proteins. (c) Functional classification of Masc-specific interacting proteins. (d) STRING protein–protein interaction network of the 52 common interactors. Nodes represent proteins and edges represent predicted interactions (combined score ≥ 0.15). Isolated nodes indicate proteins lacking known interactions, reflecting the current annotation status of the silkworm proteome; Figure S3: Transcriptomic analysis of female-sterile *Bmdsx* mutants. (a) Venn diagram of dysregulated genes in *Δdsx^F^*-F and *Δdsx^C^*-F. (b) Numbers of upregulated and downregulated genes. (c,d) GO enrichment analysis of upregulated (c) and downregulated (d) genes. (e,f) KEGG pathway enrichment analysis of upregulated (e) and downregulated (f) genes; Figure S4: Transcriptomic analysis of male-sterile *Bmdsx* mutants. (a) Venn diagram of dysregulated genes in *Δdsx^M^*-M and *Δdsx^C^*-M. (b) Numbers of upregulated and downregulated genes. (c,d) GO enrichment analysis of upregulated (c) and downregulated (d) genes. (e,f) KEGG pathway enrichment analysis of upregulated (e) and downregulated (f) genes; Table S1: Primers used in this study; Table S2: Detailed information of 52 common interacting proteins of PSI and Masc.
