# Supplementary material for: PSI/Masc-Dsx Regulatory Network in Silkworm Sex Determination Pathway
Source: Insects. 2026 Jul 20;17(7):742. doi: 10.3390/insects17070742 (PMC13411575; doi:10.3390/insects17070742)

Supplementary data

Table S1 Primers used in this study.

Primer name	Primer sequence (5′ to 3′)
M-F1	tagggaattggactagtcgctagcATGACATCGGCAAAAGTAGC
M-R1	gctgattatgatcctcgagGATCGTGACTTCCTTCTGTCGATTCACAAAGCCATTTAC
M-F2	CAGAAGGAAGTCACGATCGAAGCTAATCAGAAGAAAAT
M-R2	gctgattatgatcctcgagCTATTGAAACGGCGGTGGT
Masc-O-F	CACTGGCGGCGACAAGAGACGTCATGACATCGGCAAAAGTAGC
Masc-O-R	GATTATGATCTAGAGTCGGGCCCCTATTGAAACGGCGGTGGT
PSI-O-F	CACTGGCGGCGACAAGAGACGTCATGgattacaaggatgacgacgataaggattacaaggatgacgacgataagATGAGTGATTATTCTTCTATG
PSI-O-R	GATTATGATCTAGAGTCGGGCCCTCATTTTGAGTTCACTGCTGGT
Bmdsx-F-RT	AACCATGCCACCACTGATACCAAC
Bmdsx-R-RT	GCACAACGAATACTGCTGCAATCG
Bmrp49-F	TCAATCGGATCGCTATGACA
Bmrp49-R	ATGACGGGTCTTCTTGTTGG
Masc-Q-F	ATGACATCGGCAAAAGTAGC
Masc-Q-R	ATGATGACGCGGTTATCTTC
PSI-Q-F	CCACAACATCAAGACCCTTC
PSI-Q-R	GCTCCAGTTTGTTCTTGGAG

Table S2 Detailed information of 52 common interacting proteins of PSI and Masc.

Functional category	Functional annotation	Protein name	UniProt ID
Energy metabolism (10)	Mitochondrial respiratory chain Complex I core subunit (Essential gene; "lethal" mutant phenotype)	Lethal(3)neo18 / NADH dehydrogenase subunit 5	B5A989
	Mitochondrial respiratory chain Complex IV subunit	Cytochrome c oxidase subunit 2	D5LQX6; A0A0U1Z013; A0A0U1YZZ0; A0A0U1Z1Y3; A0A0U1YWU9; Q9MID9; Q9MM50
	Glycolysis enzyme	Enolase	A0A1L7P084; A1YQ87
Cytoskeleton (3)	Major component of microtubules	Tubulin beta chain	O01676; H9IWQ1
	Muscle structure protein	Myofilin variant B	C3UZ73
Structure protein (2)	Structural constituent of cuticle	Putative cuticle protein	C0H6J4; C0H6L9
Signal transduction (1)	Cell cycle-related kinase	Cdc2-related kinase	O17508
Gene regulation (2)	Chromatin remodeling complex subunit	Rsf1	Q1HDZ3; H9J6R3
Unknown function (34)	Function uncharacterized	Uncharacterized protein	H9IUV1; H9IWX2; H9J4L6; H9JXH0; H9J2F1; H9J7K1; H9JL27; H9JWS8; H9JVZ2; H9JF91; H9JB82; H9JSI0; H9JSD3; H9JTW5; H9JBE9; H9JR61; H9JUJ8; H9JM71; H9JH96; H9J8J5; H9J6Y6; H9J5Q7; H9J559; H9J1Q1; H9J049; H9IZC6; H9IY97; H9IXZ0; H9IXG9; H9IWX1; H9IVF8; H9ISW3; H9JHG2

Figure S1 Validation of overexpression in cultured cells. (a) RT-PCR analysis of *Bmdsx* splicing patterns in the indicated cell samples. (b, c) Relative expression levels of *BmPSI* (b) and *BmMasc* (c) in the indicated cell samples, as determined by qRT-PCR. Statistical significance: *P < 0.05, **P < 0.01; ns, not significant. Abbreviations: Control, empty-vector-transfected cells; Blank, untransfected cells; *BmMasc-wt*, wild-type *Masc* overexpression; *BmMasc-m*, mutant *Masc* overexpression; *BmPSI*, *PSI* overexpression; *BmPSI/Masc-m*, co-overexpression of *PSI* and mutant *Masc*.

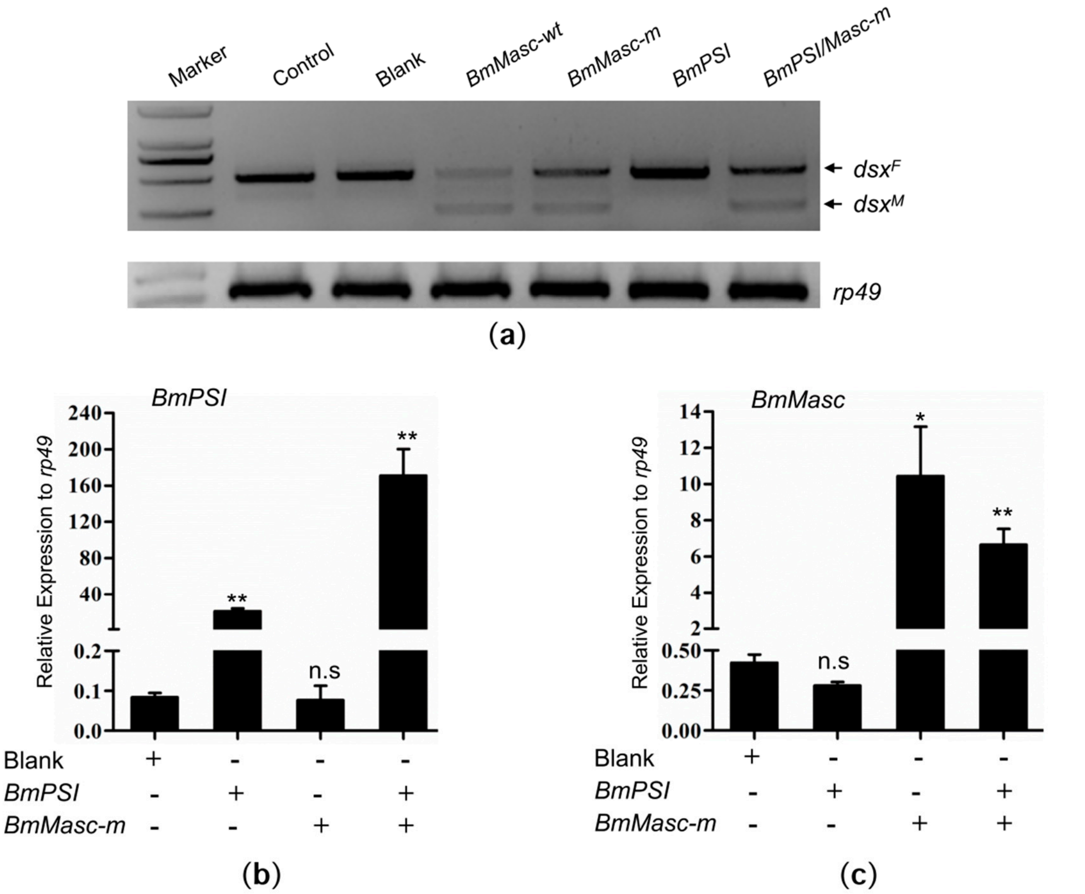

Figure S2 Functional classification of PSI- and Masc-specific interacting proteins. (a) Venn diagram of proteins identified by FLAG pull-down. (b) Functional classification of PSI-specific interacting proteins. (c) Functional classification of Masc-specific interacting proteins. (d) STRING protein-protein interaction network of the 52 common interactors. Nodes represent proteins and edges represent predicted interactions (combined score ≥ 0.15). Isolated nodes indicate proteins lacking known interactions, reflecting the current annotation status of the silkworm proteome.

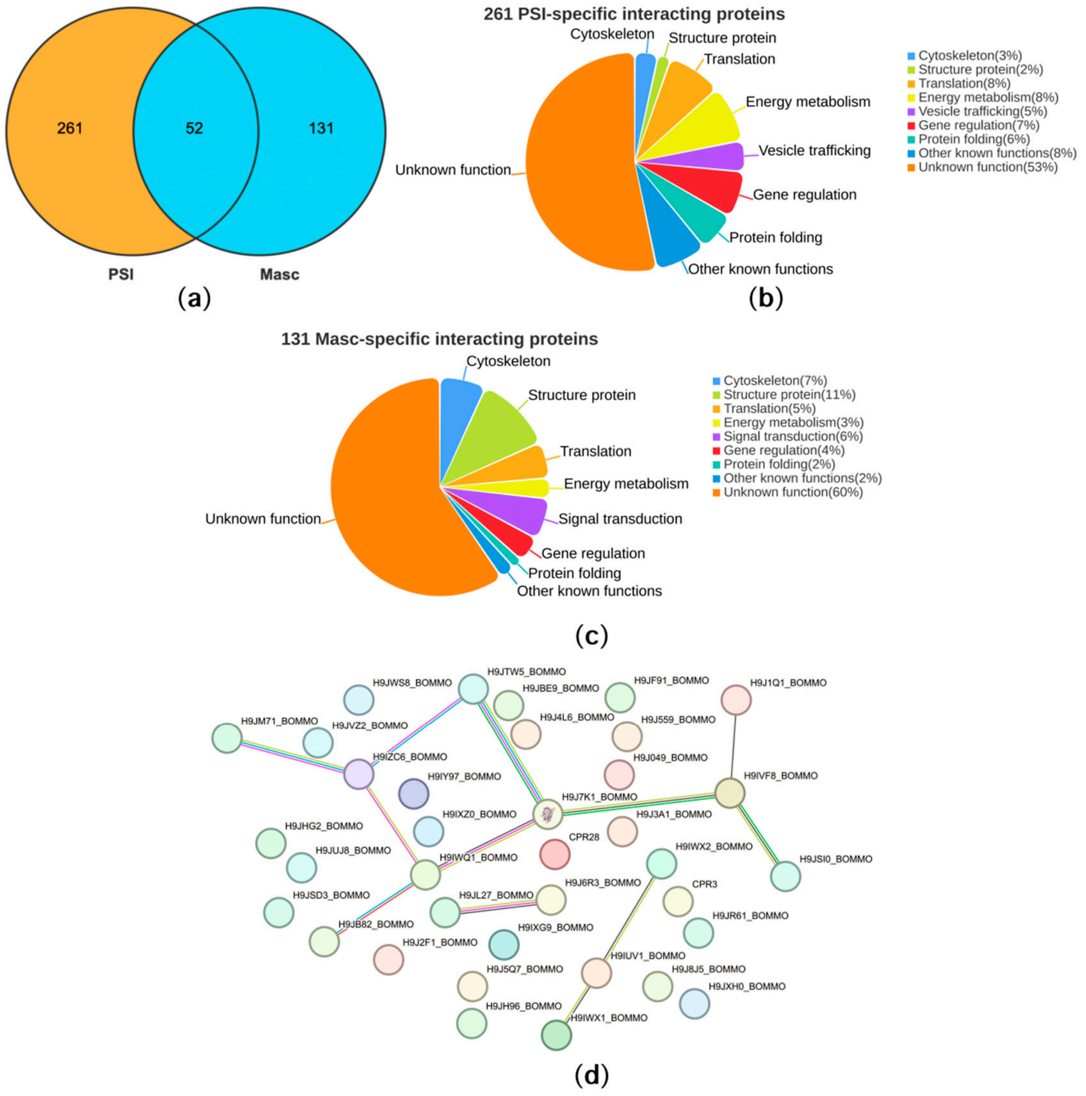

Figure S3 Transcriptomic analysis of female-sterile *Bmdsx* mutants. (a) Venn diagram of dysregulated genes in *Δdsx*^{F}*-F* and *Δdsx*^{C}*-F*. (b) Numbers of upregulated and downregulated genes. (c, d) GO enrichment analysis of upregulated (c) and downregulated (d) genes. (e, f) KEGG pathway enrichment analysis of upregulated (e) and downregulated (f) genes.

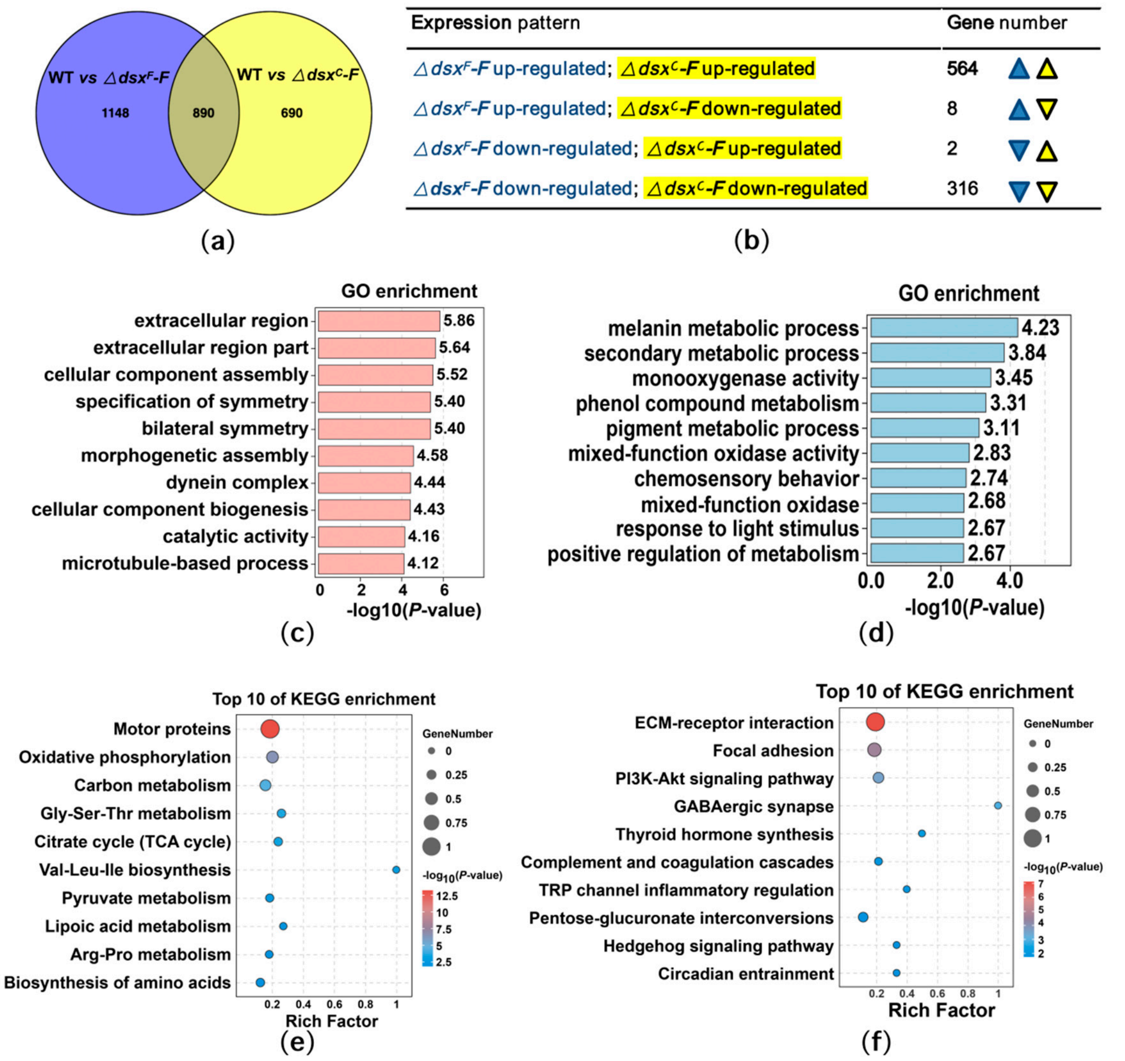

Figure S4 Transcriptomic analysis of male-sterile *Bmdsx* mutants. (a) Venn diagram of dysregulated genes in *Δdsx^{M}-M* and *Δdsx^{C}-M*. (b) Numbers of upregulated and downregulated genes. (c, d) GO enrichment analysis of upregulated (c) and downregulated (d) genes. (e, f) KEGG pathway enrichment analysis of upregulated (e) and downregulated (f) genes.

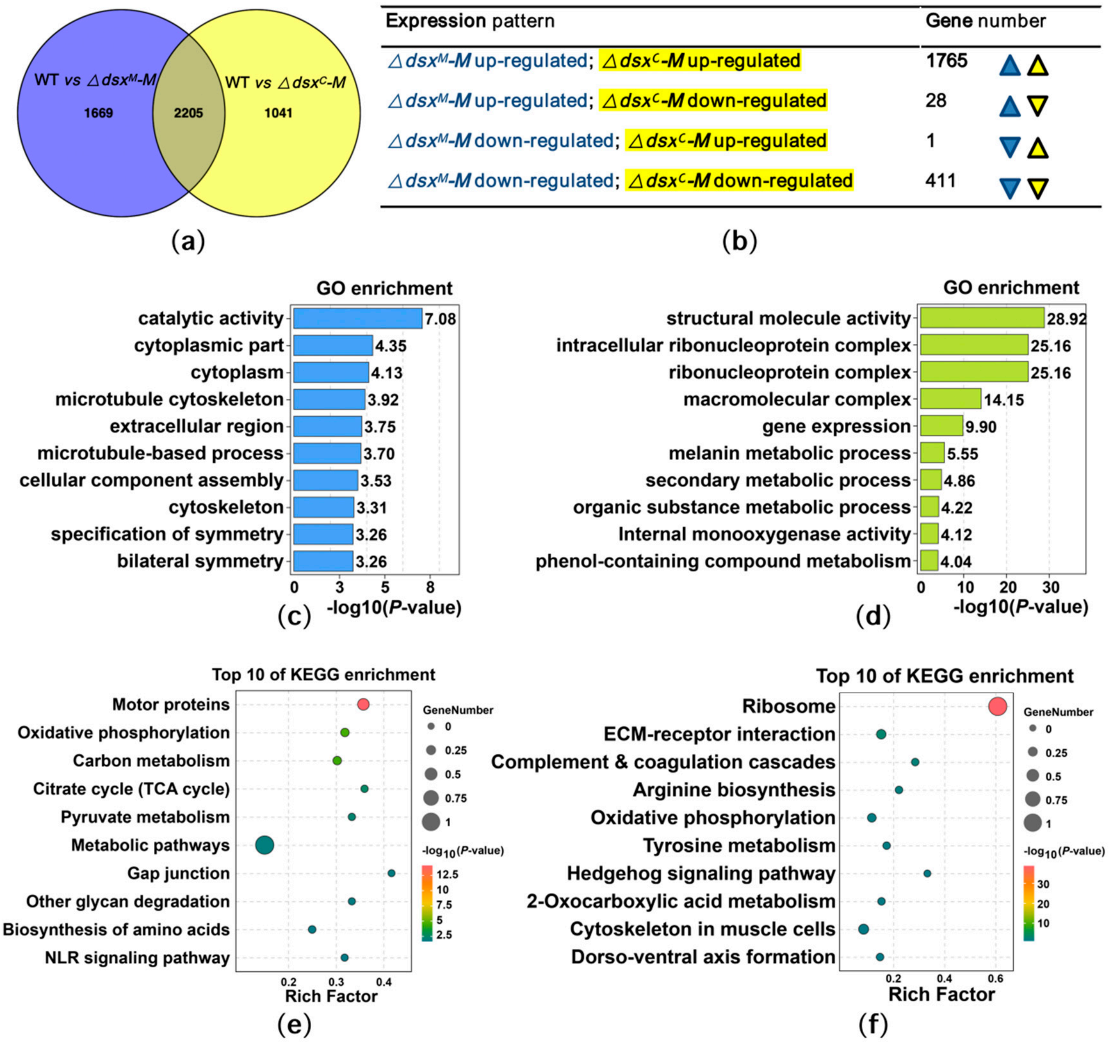

Supplement: Supplementary file 1 [file insects-17-00742-s001.zip › insects-4363022-supplementary.pdf]
